# Atomic-scale Chemical Imaging and Quantification of Metallic Alloy Structures by Energy-Dispersive X-ray Spectroscopy

**DOI:** 10.1038/srep03945

**Published:** 2014-02-04

**Authors:** Ping Lu, Lin Zhou, M. J. Kramer, David J. Smith

**Affiliations:** 1 Sandia National Laboratories, PO Box 5800, MS 1411, Albuquerque, NM 87185-1411 USA; 2 Ames Laboratory, Ames, IA 50014 USA; 3 Department of Physics, Arizona State University, Tempe, AZ 85287 USA

## Abstract

Determination of atomic-scale crystal structure for nanostructured intermetallic alloys, such as magnetic alloys containing Al, Ni, Co (alnico) and Fe, is crucial for understanding physical properties such as magnetism, but technically challenging due to the small interatomic distances and the similar atomic numbers. By applying energy-dispersive X-ray spectroscopy (EDS) mapping to the study of two intermetallic phases of an alnico alloy resulting from spinodal decomposition, we have determined atomic-scale chemical composition at individual lattice sites for the two phases: one is the B2 phase with Fe_0.76_Co_0.24_ -Fe_0.40_Co_0.60_ ordering and the other is the L2_1_ phase with Ni_0.48_Co_0.52_ at A-sites, Al at B_Ι_-sites and Fe_0.20_Ti_0.80_ at B_ΙΙ_-sites, respectively. The technique developed through this study represents a powerful real-space approach to investigate structure chemically at the atomic scale for a wide range of materials systems.

Intermetallic magnetic alloys, containing Al, Ni, Co (therefore, alnico) and Fe represent a class of nanostructured functional alloys that have important industrial applications^1^,^2^. These nanostructures, which are formed as a result of spinodal decomposition, typically consist of a primary Fe-Co-rich phase with dimensions on the order of several tens of nanometers, residing in a continuous Al-Ni-rich matrix. The specific structure is very sensitive to alloy chemistry as well as the crystallographic orientation of the primary phase relative to an external magnetic field imposed during decomposition^1^. Accurate determination of the crystal structure of the spinodal phases, i.e., identification of atoms and their lattice-site occupancies, is crucial for understanding the magnetic properties. However, due to coexistence of nanometer-scale phases as well as alloy compositions with atoms of similar atomic number (e.g. Fe, Co, Ni), accurate determination of the lattice structures of these phases has not been possible by traditional imaging or diffraction techniques (i.e., electron, neutron, and X-ray scattering).

Recent advances in atomic-scale chemical mapping in scanning transmission electron microscopy (STEM) provide an opportunity to determine directly the crystal lattice *and* chemical structure of the intermetallic phases. With the development of spherical aberration correction technology for electron microscopy, it has been demonstrated that atomic-scale chemical mapping can be obtained by scanning an Ångstrom-sized electron probe across a sample and collecting either electron-energy-loss spectra (EELS; thus STEM-EELS)^3^,^4^,^5^,^6^,^7^,^8^ or energy-dispersive X-ray spectra (EDS; thus STEM-EDS)^9^,^10^,^11^,^12^,^13^,^14^,^15^. Simultaneously, electrons which have been scattered through large angles after exiting the specimen are also used to record Z-contrast or high-angle annular dark-field (HAADF) images that provide a reference image for location of atomic columns. These efforts have so far mostly been applied to materials such as perovskite oxides^5^,^6^,^7^,^8^,^9^,^13^,^15^,^16^, e.g. SrTiO_3_, and compound semiconductors^10^,^11^, which have well-known crystal structures with relatively large lattice spacings, that are also relatively resistant to electron-beam irradiation. This radiation-resistant property allows relatively long dwell times at each recording point (i.e., pixel) for enhancing the signal-to-noise (S/N) ratio that is necessary for elemental mapping. These requirements have, however, severely limited the materials that can be studied by chemical mapping techniques. In particular, there are no reports thus far on atomic-scale EDS studies of metallic alloy structures using these techniques, which is presumably due both to their smaller lattice spacings and their electron beam sensitivity. The absence of such reports points to the necessity for a technique with better spatial resolution and reduced beam damage.

In this paper, we report the crystal structure of alnico intermetallic alloys obtained using the latest atomic-scale EDS mapping methods. By improving the spatial resolution between identical atom columns in STEM-EDS mapping to better than 2 Å, and with observations in multiple crystallographic directions, we have determined unambiguously the lattice structures of two intermetallic phases formed via spinodal decomposition. The structural information obtained from this study facilitates a better understanding of the magnetic properties of these alloys. The technique developed through this study also provides a powerful real-space approach to investigate structure chemically at the atomic scale and should prove to be highly useful for investigating other more complex systems.

The microstructure of the alnico alloy is shown in Fig. 1a, and consists of the magnetic Fe-Co-rich (α_1_) phase and the Al-Co-Ni-rich matrix (α_2_). The alnico has an average composition of Fe-33.7Co-31.8Ni-11.8Al-13.8Ti-5.5Cu-2.7 in atomic percentages, as measured by inductively coupled atomic emission spectroscopy (see supplementary Table S1). The relevant crystal structures for the α_1_ and α_2_ phases are shown in Fig. 1b: (i) a body-centered-cubic (BCC) with the same types of atoms occupying both lattice sites at the corner (A-sites) and at the body-center (B-sites); (ii) a B2 structure with A-sites and B-sites occupied by different atoms and (iii) a L2_1_ structure, which displays two types of B2 structure (with B_Ι_ and B_ΙΙ_ sites, respectively) placed in a specific order which results in a cubic unit cell that is doubled along x-, y-, and z-directions. The α_1_ phase is either BCC or B2 phase, depending on whether the Fe and Co atoms occupy the A- or B-sites randomly (a disordered structure) or occupy the A- and B- sites preferentially (an ordered structure). Due to the similar electron scattering powers of Fe and Co atoms, the two structures (BCC and B2) cannot be distinguished based solely on HAADF images such as shown in Fig. 1c. The α_2_ phase, on the other hand, has the L2_1_ structure, and in this case due to overlapping of the B_Ι_ and B_ΙΙ_ sites (shown as blue and green atoms in Fig. 1c(iii), respectively) in the [001] direction, the HAADF image (Fig. 1d) exhibits a smaller (1 × 1) unit cell, which is half the dimensions of the actual cell. To resolve atoms at the B_Ι_ and B_ΙΙ_ sites requires imagining of the structure along the [110] direction.

## Results and Discussion

### Improvement of EDS spatial resolution

In chemical mapping, the shortest lateral distance between columns of identical atoms in a crystal projection determines the required spatial resolution necessary for resolving the atomic columns^16^. The required spatial resolution is 3.9 Å for SrTiO_3_ in the [001] direction and about 3.5 Å for GaAs in the [110] direction. The smaller dimensions of the unit cell (~2.9 Å) for BCC intermetallic alloys require improving the spatial resolution in order to resolve the structure chemically. We show here that this can be achieved by a combination of small electron probe (<1.5 Å), using a thin TEM specimen (<20 nm) and improving of the S/N ratio through averaging of EDS maps that are related to each other via lattice-vector translations in the image plane (for convenience, we have termed this process “lattice-averaging”, see supplementary Figure S1). For thin TEM specimens, the EDS signal is localized to atomic columns^5^,^15^. Furthermore, under such conditions, EDS X-ray counts from individual atomic columns can be approximated by a Gaussian distribution and thus be properly integrated by fitting Gaussian peaks at each atomic column position^15^,^16^; the chemical composition at the atomic column positions can then be calculated column-by-column using the Cliff-Lorimer method^17^. The width of the Gaussian peak, defined as the full-width at half-maximum (FWHM), which ultimately determines the spatial resolution of the chemical mapping, is effectively determined by convoluting the electron probe with the effective EDS local ionization potential under thin specimen conditions. By selecting a small electron probe (<1.5 Å) and an appropriately thin area on the specimen, the spatial resolution of chemical mapping can be improved. The specimen thickness for the approximation to be valid must be limited to be less than ~20 nm. The selection of small electron probe and thin specimen leads, however, to significant reduction in X-ray count. This reduction can be compensated by lattice-averaging EDS maps that are related by lattice translation vectors, effectively improving the EDS counting statistics (see supplementary Figure S1).

### Determination of α_1_ phase structure

Figs. 2a and 2b show EDS maps of Fe K_α_ and Co K_α_, respectively, obtained from the α_1_ phase in the [001] projection. The maps were lattice-averaged sixteen times by translating the map across the region using 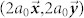 as the basic translation vectors, where a_o_ equals 2.9 Å (i.e. the unit-cell constant of the BCC cell), and 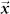 and 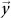 are the unit vectors in x-, and y- directions. The lattice averaging improves the S/N significantly, and clearly allows direct visualization of atoms at the lattice sites. Fig. 2c shows a color map of the Fe K_α_ (red) and Co K_α_ (green). Ordering takes place in this structure with the Fe and Co atoms preferentially occupying the A-sites or B- sites, respectively. Moreover, the thin specimen condition allows the X-ray counts from each atomic column to be integrated through the fitting of Gaussian peaks at the atomic column positions^15^. Fig. 2d shows a 3-dimensional (3-D) surface plot of the experimental (red) and fitted (green) maps for the Fe K_α_. The fitting allows the X-rays from the individual column to be properly counted. Similar fitting was also performed for the Co K_α_ map. The experimental EDS map and the Gaussian peak-fitted map are further shown as line profiles along the [100] direction in Fig. 2e. Note that Fe and Co atoms are present at both A- and B-sites, but Fe atoms preferentially occupy the A-sites, while Co atoms preferentially occupy the B-sites. The x-ray counts for the atomic sites are obtained by integrating Gaussian peak-fitted peaks located individually at the atomic sites. With the X-ray counts from the atomic columns known, one can simply proceed to calculate the chemical composition at each column site using the Cliff-Lorimer method^17^. The A- and B-sites for the α_1_ phase were found to have averaged atomic compositions of Fe_0.76_Co_0.24_, and Fe_0.40_ Co_0.60_, respectively (for an example of the calculation, see supplementary Table S2). The FWHM of the Gaussian peaks for CoK_α_ and Fe K_α_ is determined to be 1.9 Å which is considerably smaller than 2.8 Å for Fe K_α_ reported previously for perovskite oxide^15^. The smaller peak width in these experiments is a direct result of using the smaller electron probe and the thin specimen condition.

### Determination of α_2_ phase structure

**Figure 1:**
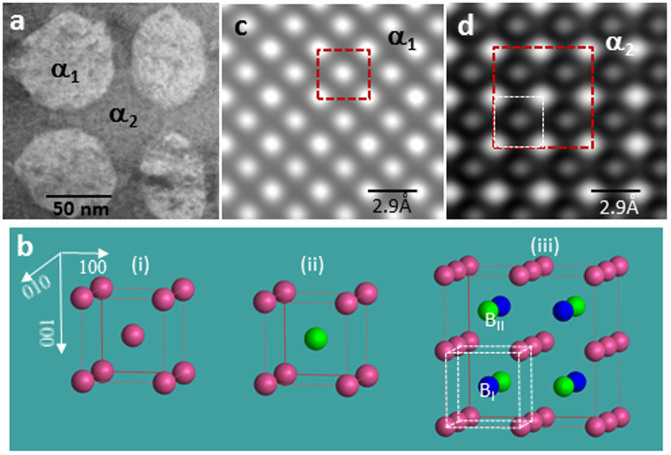
(a) HAADF image of alnico alloy showing composite structure consisting of isolated Fe-Co-rich (α_1_) phase (lighter contrast) embedded in continuous Al-Co-Ni-rich (α_2_) phase; (b) models of three crystal structures: (i) BCC; (ii) B2 and (iii) L2_1_; and (c) and (d): high-resolution HAADF image of α_1_ and α_2_ phase, in [001] direction, respectively. In the model of the L2_1_ structure, there are two kinds of B sites, i.e. blue and green atoms, marked as B_I_ and B_II_, respectively.

**Figure 2:**
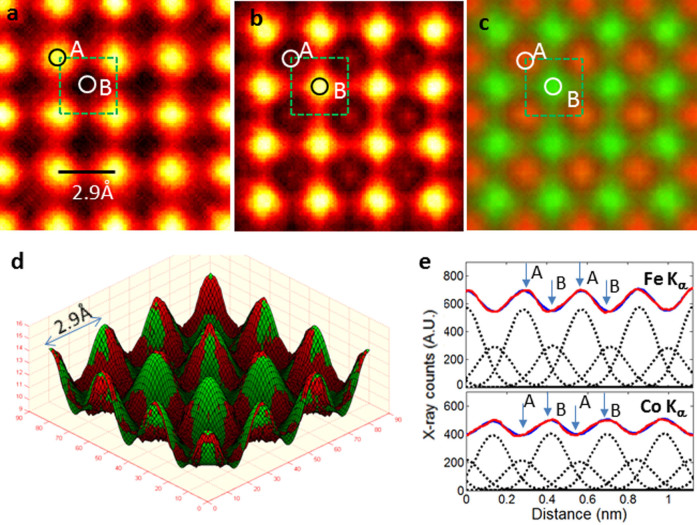
(a) EDS map of Fe K_α_; (b) EDS map of Co K_α_; (c) composite color map of Fe K_α_ (red) and Co K_α_ (green); (d) surface-plot of experimental (red) and the least-square Gaussian-fitted (green) Fe K_α_ map; and (e) line profiles of EDS X-ray counts for Fe K_α_ and Co K_α_ along [100] direction. Maps obtained from α_1_ phase in [001] direction. In (e), the solid blue line is the experimental data; the solid red line is from the least-square Gaussian-fitting; the individual Gaussian peaks at the lattice positions from the fitting are shown as dashed black lines; and arrows marked A and B indicate positions of A- and B- sites along [100] direction. Lattice site positions (A, B) and size of unit cell in [001] projection are also marked by circles and dashed square in EDS maps in (a), (b) and (c).

**Figure 3:**
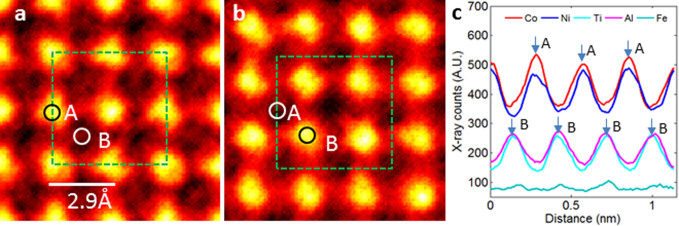
(a) EDS map of combined Co K_α_ and Ni K_α_; (b) EDS map of combined Al K_α_, Ti K_α_ and Fe K_α_; and (c) line profiles of Co K_α_, Ni K_α_, Al K_α_, Ti K_α_ and Fe K_α_ along [100] direction. The maps are obtained from α_2_ phase in [001] direction. Line-profiles in (c) indicate Co and Ni atoms at A-sites and Al, Ti and Fe at B-sites, or B_I_ and B_II_ sites, which overlap in [001] direction. Lattice site positions (A, B) along [100] direction are marked by arrows in (c). Dashed squares in (a) and (b) mark size of L2_1_ unit cell in [001] projection, and circles in (a) and (b) mark positions of A-sites and B-sites (or B_I_ and B_II_ sites, overlapped).

**Figure 4:**
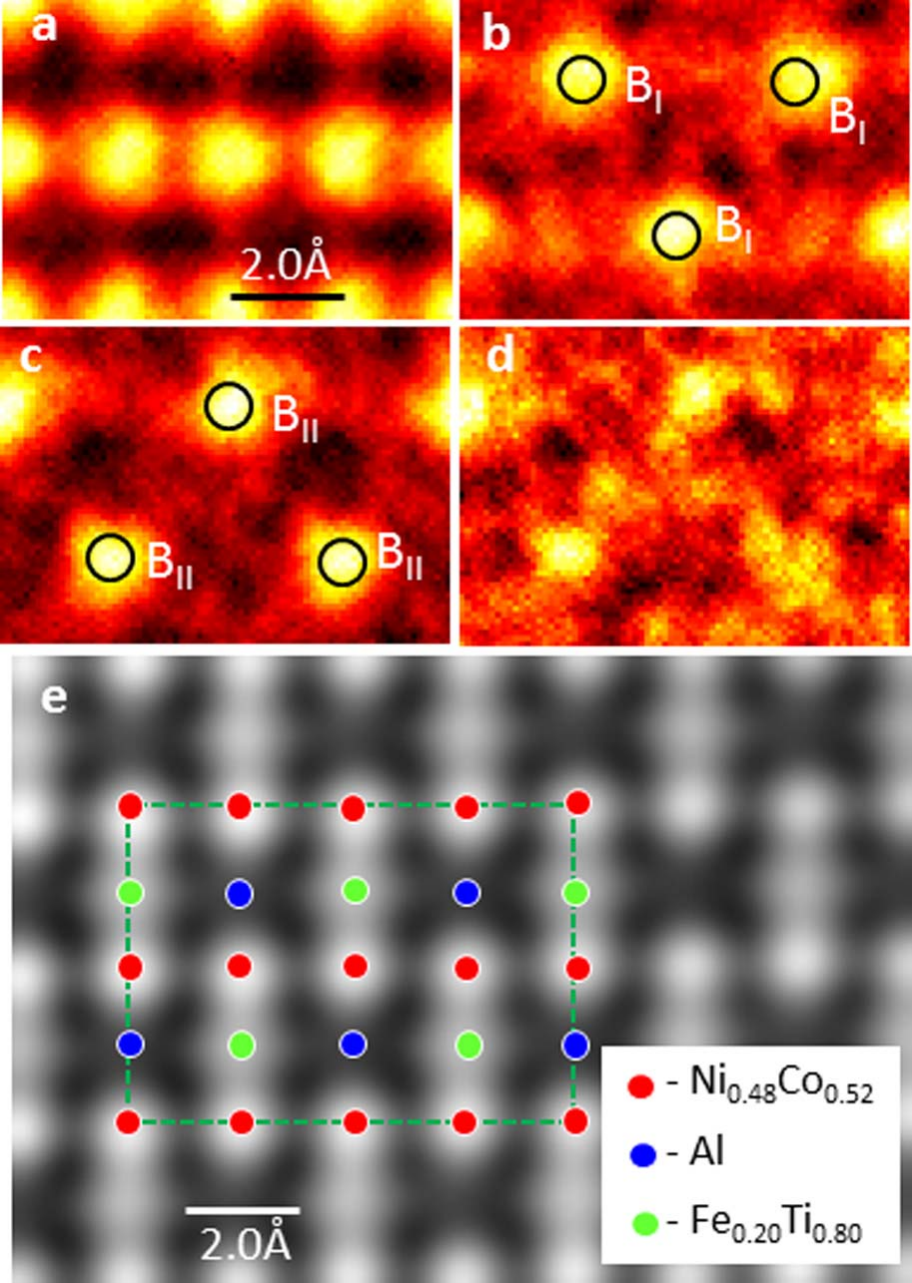
EDS maps of α_2_ phase in [110] direction. (a) Co/Ni K_α_; (b) Al K_α_; (c) Ti K_α_ and (d) Fe K_α_. (e) high-resolution HAADF image of α_2_ phase in [110] direction. Positions of B_I_ and B_II_ sites in [110] projection are marked by circles in (b) and (c). Quantified structure for α_2_ phase is shown in superposition with HAADF image in (e).

The α_2_ phase was imaged in both the [001] and [110] directions in order to determine its structure. Fig. 3a shows the EDS map in the [001] direction combining X-rays from both Co K_α_ and Ni K_α_. The combined EDS map for Al K_α_, Ti K_α_ and Fe K_α_ X-rays is shown in Fig. 3b. The maps were obtained after lattice-averaging over nine positions related by the basic translation vectors 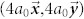. The basis for combining the different X-rays is shown in Fig. 3c, which shows the x-ray line profiles along the [100] direction for five different materials (Co K_α _, Ni K_α _, Ti K_α _, Al K_α_ and Fe K_α _). It is clear that Co and Ni atoms together occupy the A-site, and Al, Ti and Fe atoms occupy the B_I_ or B_II_ sites in the L2_I_ structure. Since the B_I_ and B_II_ sites of the L2_I_ structure overlap in the [001] direction (Fig. 1b(iii)), it is not possible to resolve individually the atoms (among Al, Ti and Fe) at the B_I_ and B_II_ sites based on mapping along the [001] direction. Fig. 4 shows EDS maps for Co/Ni K_α_, Al K_α_, Ti K_α_ and Fe K_α_, together with the HAADF image in the [110] direction. These maps were obtained after lattice-averaging eight times using translation vectors 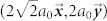. In the [110] direction, the B_I_ and B_II_ sites can be resolved directly. It is clear from these maps that Al atoms and Ti atoms are located at B_I_ and B_II_ sites, respectively. The map for Fe K_α_ (Fig. 4d) is very noisy due to the relatively small overall concentration (<5 at%) of Fe in the α_2_ phase. Nevertheless, the Fe atoms preferentially stay with the Ti atoms (or B_II_ sites). Note that the Co/Ni K_α_ map (Fig. 4a) clearly resolves the Co/Ni columns, confirming the spatial resolution of the chemical map is better than 2 Å.

With the information obtained from the chemical mapping in the [110] (Fig. 4), the chemical compositions at the lattice sites for the α_2_ phase can be calculated using the EDS maps in the [001] direction. Following the procedure used to fit the maps with individual Gaussian peaks at the lattice sites, the chemical composition has been calculated column-by-column using the X-ray intensity obtained from the fitted Gaussian peaks. We have determined that the average chemical composition is Ni_0.48_Co_0.52_ at the A-sites, Al at the B_I_-sites and Fe_0.20_Ti_0.80_ at the B_II_-sites, respectively (see supplementary Table S2). In Fig. 4e, the structure model for the α_2_ phase is shown in superposition with the HAADF image in the [110] projection. To test the reasonableness of these results, the element concentration in the bulk alnico alloy was calculated from the site concentrations determined by this study and found to be consistent with measured alloy composition (see supplementary Table S1). Furthermore, the B2 ordering of the dominantly Fe-Co phase and the site occupancies of the L2_1_ phase have each been reported separately in bulk samples near these respective compositions^18^,^19^. In summary, we demonstrate a chemical mapping technique capable of determining atomic-scale chemical structure of individual phases within a complex nanostructured alloy.

## Conclusions

We have applied atomic-scale STEM-EDS techniques to investigate two intermetallic phases of an alnico alloy that resulted from spinodal decomposition. The chemical compositions were determined at individual lattice sites for the two phases: one as B_2_ phase with Fe_0.76_Co_0.24_ -Fe_0.40_Co_0.60_ ordering and other as L2_1_ phase with Ni_0.48_Co_0.52_ at A-sites, Al at B_I_-sites and Fe_0.20_Ti_0.80_ at B_II_-sites. The structural details of the α_1_ and α_2_ phases obtained from this study provide crucial information for understanding the magnetic properties of the alloy.

The technique developed in this study represents a powerful approach to evaluate crystal structure chemically in real space at the atomic scale, and should help to unravel the often complicated relationships between the structural, chemical and physical properties of complex materials. Through the combination of a small electron probe, thin TEM specimen condition and improved S/N ratio via lattice-averaging of EDS elemental maps, we have shown that the spatial limit of chemical resolution in EDS mapping can be improved to better than 2 Å. This improvement opens up the possibility for direct atomic-scale mapping of crystal structures for a wide range of materials in multiple crystallographic projections.

## Methods

### STEM-EDS spectral imaging mapping

A FEI Titan™ G2 80–200 STEM with a Cs probe corrector and ChemiSTEM™ technology (X-FEG™ and SuperX™ EDS with four windowless Si drift detectors), operated at 200 kV was used in this study^12^. For atomic-scale chemical mapping, EDS spectral image data were acquired with an electron probe of size <1.5 Å, convergence angle of 18.1 mrad, and current of ~100 pA. HAADF images were recorded under similar optical conditions using an annular detector with a collection range of 60–160 mrad. Spectral images were acquired as a series of frames, where the same region was scanned multiple times. Frames were spatially drift-corrected to build up spectral image data. The instantaneous dwell time on each pixel was 100 μsec, and a typical frame was 256 × 256 pixels. Spectral image collection typically took about 1200 sec, yielding a total per-pixel dwell time of about 18 msec.

### TEM specimen preparation and thickness determination

The alnico alloy is a commercial grade alloy supplied by Arnold Magnetic Technologies Corp. The alloy is directionally casted with most grains aligned along the [001] direction, then isothermally annealed with an applied magnetic field along the casting direction. The TEM specimen used for this study was mechanically polished to a thickness of less than 1 μm, followed by short-time low voltage Ar ion-milling with liquid nitrogen cooling. To limit oxide formation, the TEM sample was kept under vacuum and examined within 48 hrs after its preparation. Thin specimen areas with thicknesses less than 20 nm were used for high-resolution EDS mapping. The TEM specimen thickness was estimated based on the EDS count rate. Specifically, a Cu nanoparticle sphere with a diameter of 12 nm was used as a calibration standard. The electron probe was incident on top of the Cu sphere, and an EDS spectrum was collected for a fixed time of 50 sec. Another EDS spectrum was collected from the alnico TEM sample under identical experimental conditions. The X-ray count for the alnico sample was converted to the Cu K_α_ using the proper k-factors for each element. The ratio of the Cu K_α_ X-ray counts between the two spectra was used to determine the sample thickness.

### Gaussian peak-curve fitting

Microsoft (Microsoft Corporation, Redmond, WA) Excel 2010 with the “Solver” application, which is embedded in the “Analysis ToolPak”, was used for constrained nonlinear optimization. The “Solver” allows easy and flexible linear and nonlinear optimization to functions defined in the spreadsheet. During fitting, the positions of Gaussian peaks were fixed at the lattice positions (A-, and B-sites) and the width of the Gaussian peak at the lattice sites was constrained to be equivalent from column to column, while the peak heights and peak widths were allowed to vary.

## Author Contributions

P.L. performed the EDS experiments, interpreted the data and wrote the paper. L.Z. provided the TEM sample, carried out the initial TEM work on the sample and contributed to the paper writing. M.J.K. and D.J.S. contributed to the discussion and writing and preparation of the manuscript.

## Supplementary Material

Supplementary Information Supplementary Information
