# Supplementary material for: Atomic-scale Chemical Imaging and Quantification of Metallic Alloy Structures by Energy-Dispersive X-ray Spectroscopy
Source: Sci Rep. 2014 Feb 4;4:3945. doi: 10.1038/srep03945 (PMC3912478; doi:10.1038/srep03945)
Supplement: Supplementary Information [file srep03945-s1.pdf]

Supplementary Information

Atomic-scale Chemical Imaging and Quantification of Metallic Alloy Structures by Energy-Dispersive X-ray Spectroscopy

Ping Lu[1*], Lin Zhou[2], M. J. Kramer[2], and David J. Smith[3]

[1]Sandia National Laboratories, PO Box 5800, MS 1411, Albuquerque, NM 87185-1411 USA

[2]Ames Laboratory, Ames, IA 50014 USA

[3]Department of Physics, Arizona State University, Tempe, AZ 85287 USA

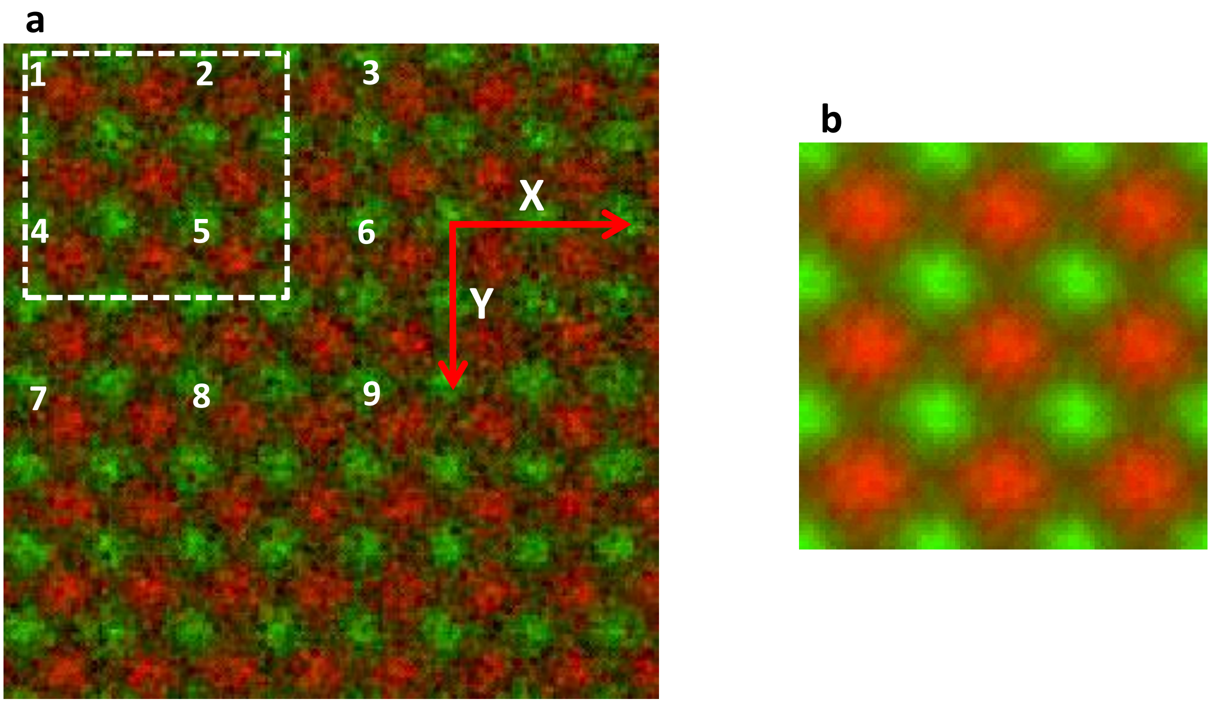

Figure S1. Method of the lattice averaging used to improve the signal-to-noise (S/N) ratio of X-ray mapping: (a) a raw X-ray map extracted from EDS spectral imaging; and (b) lattice-averaged X-ray map. In (a), a smaller EDS region, size of 3x3 unit cells (marked by dashed square) is chosen, maps of this region from nine equivalent positions (marked by 1, 2, …9) in the image,

defined by using basic lattice translations vectors (**X**, **Y**), are averaged to obtain the lattice-averaged X-ray map in (b). In this particular example, **X**= $2a_0\vec{\boldsymbol{x}}$, and **Y**= $2a_0\vec{\boldsymbol{y}}$ are chosen as basic translation vectors, where a_o is the unit cell constant and $\vec{\boldsymbol{x}}$ and $\vec{\boldsymbol{y}}$ are the unit vectors in x-, and y- directions.

(at%)	Fe	Co	Ni	Al	Ti	Cu
**calculated**	30	34	12	13	10	
**measured**	33.7	31.8	11.8	13.8	5.5	2.7

Table S1. Calculated bulk alloy concentration from EDS atomic-scale chemical mapping and measured bulk alloy composition by inductively coupled plasma atomic emission spectroscopy. The calculation is based on estimation of α_1 phase's volume fraction (ρ) by the ratio of maximum remenance (B_r) and saturation magnetization of FeCo alloy ($M_s^{Fe,Co}$) : $B_r = \rho M_s^{Fe,Co}$ [1] and assuming complete segregation of Co and Fe from the $L2_1$ phase and segregation of the Cu to a separate grain boundary phase. Here we choose 2.2T for the saturation magnetization of FeCo alloy [2]. The measured B_r for alnico 9 is 1.06T, thus, $\rho=1.06/2.2=0.48$. Then, the volume fraction of α_2 phase is $1- \rho$. The atomic concentration of each element in the bulk alloy can be calculated from site occupancy measured by EDS. For example, the Fe concentration can be calculated by: $C_{Fe} = 0.48 \times (0.76 + 0.4) \times 0.5 + (1 - 0.48)(0.2 \times 0.25) = 0.30$. Table S1 showed good agreement between calculation and measured bulk concentration, which indicates the accuracy of our EDS technique. The relatively larger discrepancy between Ti and Fe elements may due to oxidation of Fe and Ti at the TEM sample surface. The measured concentration for α_2 phase is also within the thermal dynamically stable range for $L2_1$ phase from Fe-Ti-Al and Al-Ti-Co phase diagrams [3].

α1-phase			α2-phase	
A site	$Fe_{0.76 \pm 0.018}Co_{0\cdot24\pm0.018}$		A site	$Ni_{0.48\pm0.025}Co_{0.52\pm0.025}$
B site	$Fe_{0.40\pm0.024}Co_{0.60\pm0.024}$		B_I Site	Al
			B_{II} Site	$Fe_{0.20\pm0.016}Ti_{0.80\pm0.016}$

Table S2. Calculated chemical compositions of the lattice sites for the α_1 and α_2 phases using the Cliff-Lorimer method [4]. Example of the calculation for A-site composition in the α_1 phase is given here: I_{Fe} = 11300 (the averaged Fe Kα X-ray count at A-site, obtained by integrating the fitted Gaussian peak for Fe Kα); I_{Co} = 3786 (the averaged Co Kα X-ray count at A-site, obtained by integrating the fitted Gaussian peak for Co Kα); $k_{Fe\text{-}Co}$ =1.06 (the k-factor between Fe and Co used for this calculation); and $C_{Fe}/C_{Co} = k_{Fe\text{-}Co} \times (I_{Fe}/I_{Co})$ equation (1) and $C_{Fe} + C_{Co} = 1.0$ equation (2). $C_{Fe} = 0.76$ and $C_{Co} = 0.24$ are obtained by solving equations (1) and (2).

The errors in the calculation mainly arise from the counting statistics ($\Delta I/I$) and from the k-factor value variation ($\Delta k/k$). For $I > 1000$, $\Delta I/I < 0.03$, assuming ΔI equals to the square-root of I. The largest possible error is from the k-factor value uncertainty, which can be up to ~10% (i.e., $\Delta k/k=0.1$). The ± errors (δ) in chemical compositions in Table S2 were estimated using the relationship $\delta = C_1 \times C_2 \times (\Delta k/k)$ and assuming $\Delta k/k=0.1$, where C_1 and C_2 are the mean chemical compositions (atomic-fraction) for element 1 and 2, and k is the k-factor between element 1 and 2.

References

[1]. McCurrie, R.A. The structure and properties of AlNiCo permanent magnet alloy in *Ferromagnetic Materials* 3, Edited by E. P. Wohlfarth, North-Holland Publishing Company 107-188 (1982).

[2]. Kuhrt, C. and Schultz, L. Formation and magnetic properties of nanocrystalline mechanically alloyed Fe-Co and Fe-Ni. *J. Appl. Phys.*, 73:6588-6590, 1993.

[3]. http://www1.asminternational.org/asmenterprise/APD/default.aspx

[4] Cliff, G. & Lorimer, G.W. (1975). The quantitative analysis of thin specimen. *J. Microsc*. **103**, 203–207 (1975).
